# Continental scale variability of foliar nitrogen and carbon isotopes in *Populus balsamifera* and their relationships with climate

**DOI:** 10.1038/s41598-017-08156-x

**Published:** 2017-08-10

**Authors:** Andrew J. Elmore, Joseph M. Craine, David M. Nelson, Steven M. Guinn

**Affiliations:** 1 University of Maryland Center for Environmental Science, Appalachian Laboratory, Frostburg, MD 21532 USA; 2 Jonah Ventures, Manhattan, Kansas 66502 USA

## Abstract

Variation across climate gradients in the isotopic composition of nitrogen (N) and carbon (C) in foliar tissues has the potential to reveal ecological processes related to N and water availability. However, it has been a challenge to separate spatial patterns related to direct effects of climate from effects that manifest indirectly through species turnover across climate gradients. Here we compare variation along environmental gradients in foliar N isotope (δ^15^N) and C isotopic discrimination (Δ^13^C) measured in 755 specimens of a single widely distributed tree species, *Populus balsamifera*, with variation represented in global databases of foliar isotopes. After accounting for mycorrhizal association, sample size, and climatic range, foliar δ^15^N in *P. balsamifera* was more weakly related to mean annual precipitation and foliar N concentration than when measured across species, yet exhibited a stronger negative effect of mean annual temperature. Similarly, the effect of precipitation and elevation on Δ^13^C were stronger in a global data base of foliar Δ^13^C samples than observed in *P. balsamifera*. These results suggest that processes influencing foliar δ^15^N and Δ^13^C in *P. balsamifera* are partially normalized across its climatic range by the habitat it occupies or by the physiology of the species itself.

## Introduction

Spatial variation in foliar chemical traits represent phenotypic responses to environmental and genotypic factors. Because foliar chemicals regulate key biological functions, such as photosynthesis, spatial variation in foliar chemicals can also be used to understand the functioning of landscapes, including productivity and water relations of plants^1, 2^. Global gradients in foliar chemical traits, therefore, have the potential to reveal ecological controls on plant functioning. However, both intra and inter-specific variation contribute to relationships between foliar chemical traits and climate gradients^3^, and these relationships are not necessarily congruent^4^. Analysis of how individual species respond to climate gradients could provide greater insight into ecological controls on plant functioning, possibly aiding the understanding of plant response to global change.

Of particular interest to understanding plant-environment relationships are the isotopic composition of nitrogen and carbon in leaves. Foliar δ^15^N, which serves as an indicator of terrestrial N cycling^5, 6^, varies systematically between species^7–9^, mycorrhizal associations^10, 11^ and along gradients in climate^12^, and, when measured over time, can be used to infer ecosystem response to disturbance and climate change^13–16^. Local to regional-scale anthropogenic impacts on N cycling can also be important influences on foliar δ^15^N^17–20^. Mechanistically, processes that fractionate the stable isotopes of nitrogen occur to a greater extent when N availability (i.e., supply relative to demand) is high^18, 21^. Thus, over time, processes associated primarily with gaseous N loss typically increase the δ^15^N value of the remaining inorganic N pool, causing ^15^N-enrichment in foliar tissues that draw from that pool. Conversely, systems experiencing low N availability have a more conservative N cycle and do not lose as much N with low δ^15^N values. Furthermore, when N availability is low, plants depend more strongly on N fixation, atmospheric deposition, and N from mycorrhizal fungi^6^. While the δ^15^N of N passed to plants through these processes differs, it is generally depleted in ^15^N relative to the enriched pool remaining following leaching and denitrification losses^22^. Therefore, continental- to global-scale patterns in foliar δ^15^N related to climate reflect climatic control on processes that influence N availability^6, 23^, but these patterns must account for mycorrhizal association, and nevertheless, leave considerable small-grain variability related to local processes^24^.

Carbon isotope discrimination (Δ^13^C) represents an integration of the balance of CO_2_ and water fluxes in plants, and variation in Δ^13^C is controlled by both environmental and genetic factors^22, 25^. For example, there is a well-documented positive relationship between plant-moisture status and Δ^13^C across a variety of C_3_ plants e.g. refs 26–28. This pattern emerges because long-term intrinsic water-use efficiency (ratio of photosynthesis to stomatal conductance) and Δ^13^C are both influenced by C_i_/C_a_, the ratio of intercellular to ambient [CO_2_]^29^. Dry conditions lead to reduced stomatal conductance (and C_i_, the reservoir of CO_2_ available for photosynthesis) and a proportionately greater decrease in transpiration than photosynthesis^30, 31^. This relative decline in transpiration causes increased water-use efficiency and an increase in the fixation of ^13^C relative to ^12^C. Δ^13^C decreases with increasing elevation^28^, likely related to a combination of atmospheric and edaphic factors^32^, but the mechanisms are unclear and investigation continues^22, 33^.

The existence of broad relationships between foliar isotopic composition and environmental factors suggest that these foliar chemical traits index the availability of nitrogen and water to plants^22^. However, different species grown in common environments exhibit variation in foliar δ^15^N and Δ^13^C due to distinct nutrient acquisition strategies and physiological response to the environment among species^19, 22^. For example, variation in nitrogen source (e.g., NO_3_^−^, NH_4_^+^, or dissolved organic nitrogen (DON))^22^ and mycorrhizal association between species influences foliar δ^15^N^10^. Similarly, C3 plants vary in foliar Δ^13^C due to variation in carboxylation efficiency and stomatal response to environmental conditions^22^. However, it is an outstanding question if these sources of variability among species strengthen or weaken the global to continental scale variability in foliar δ^15^N and Δ^13^C imposed by climate. The assembly of species adapted to environmental conditions at any given site, might be expected to reduce the effect of climate on foliar chemical traits. Alternatively, gradients in plant lifeform along climate or elevational gradients, such as from deciduous angiosperms to evergreen gymnosperms that exhibit divergent mean foliar Δ^13^C, could strengthen effects of precipitation and elevation^28^. To advance our understanding of the relative effects of climate and plant traits (which vary by species) on foliar isotopic composition, widely distributed observations of foliar δ^15^N and Δ^13^C in a single species are required. Yet, there remains a paucity of examples where foliar δ^15^N and Δ^13^C were measured in more than a small number of specimens (e.g., 100) of the same plant species in its natural environment e.g.,^10, 19, 28^. Furthermore, because past work has analyzed foliar δ^15^N and Δ^13^C separately, the effect of N availability on Δ^13^C (e.g., through the influence of foliar nitrogen concentration on carbon assimilation) remains understudied^34^.

Here we measure 755 specimens of the widely-distributed tree species *Populus balsamifera* for foliar [N], δ^15^N and Δ^13^C. Using these data, our objective was to explore the extent to which large gradients in climate-associated variation in foliar δ^15^N and Δ^13^C observed globally e.g., refs 10, 28, are represented in measurements of a single species. Furthermore, it has been proposed that intraspecific variation in foliar traits can either correlate with broad-scale trends along climate gradients, show no variation with climate, or exhibit an intermediate response^4^. While global interspecific variation in foliar traits is widely recognized as being valuable for understanding plant-nutrient relationships^35^, few studies have sampled a sufficient number of individuals to conduct the same analysis using intraspecific variation. Because interspecific variation in plant traits is generally stronger than intraspecific variation, we hypothesize that the global variation in foliar [N], δ^15^N and Δ^13^C of *P*. *balsamifera* will exhibit a weaker response to climate than observed in global samples collected from many species. To evaluate the extent to which patterns in *P. balsamifera* chemical traits correspond to patterns in plant functioning, we subsequently used structural equation modeling to compare the effects of climate variables and foliar [N] on Δ^13^C.

## Results

### Foliar δ^15^N

**Figure 1:**
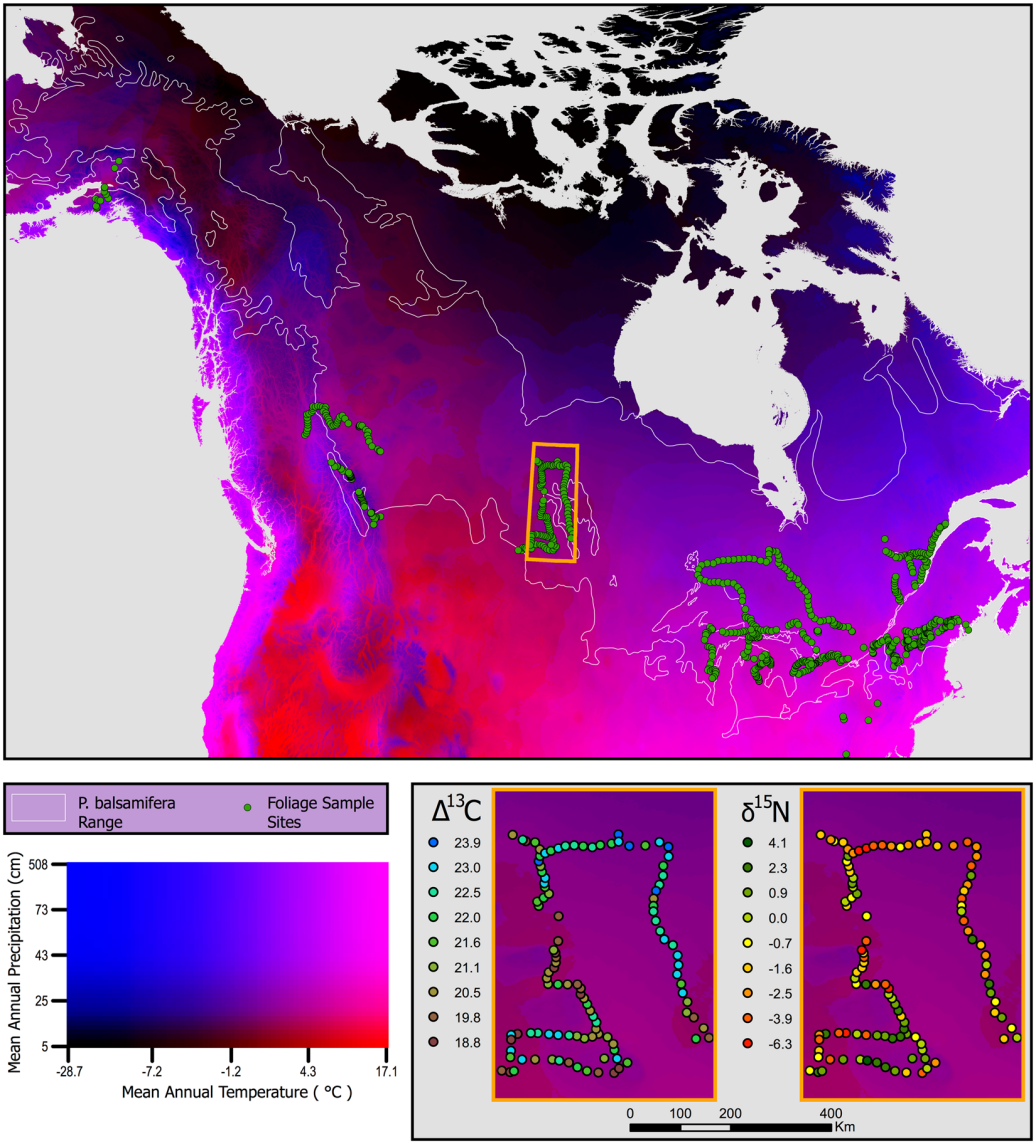
Locations of foliar *P. balsamifera* samples acquired for this study mapped over mean annual precipitation and mean annual temperature for North America. Inset shows typical fine-grain variability in foliar Δ^13^C and δ^15^N. This map of North America was produced in ArcGIS 10.2.2 (http://www.esri.com/) using Bioclim mean annual temperature and annual precipitation layers^50^. The bivariate legend was produced using custom Python code (https://www.python.org/) developed by SMG.

**Figure 2:**
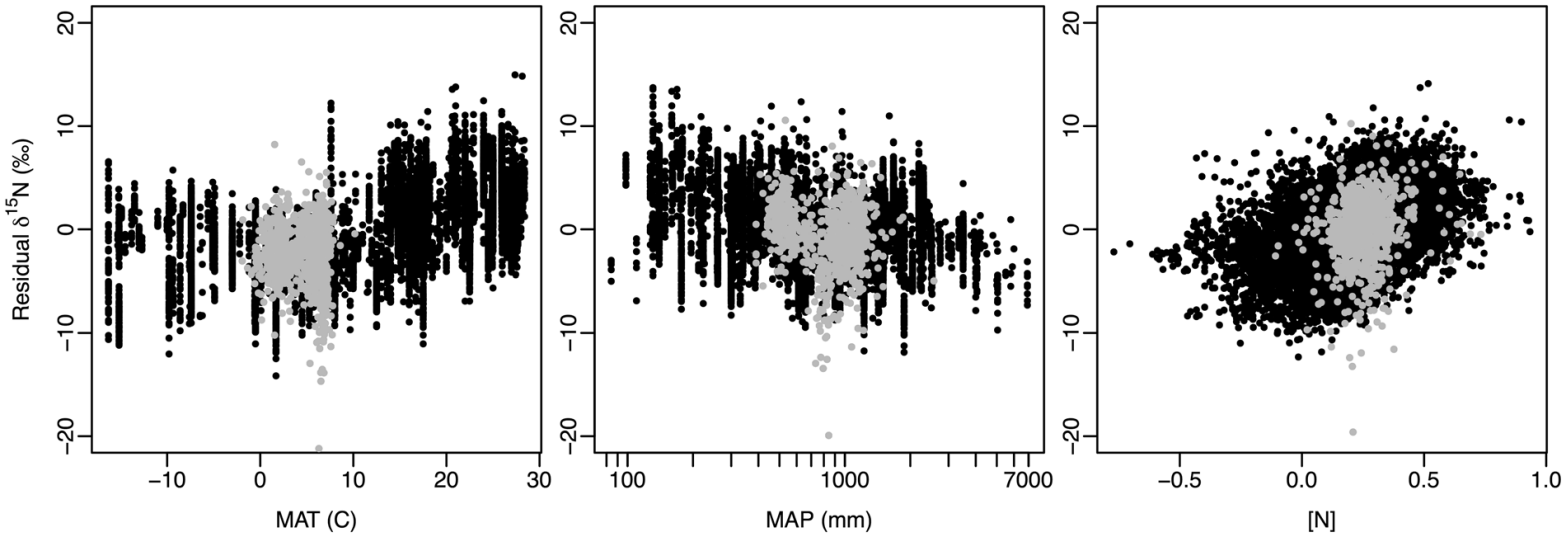
Relationship between residual δ^15^N with mean annual temperature (MAT), mean annual precipitation (log transformed MAP), and nitrogen concentration ([N]). Residual δ^15^N is calculated relative to a model including the two remaining model effects. Globally distributed observations from 1273 species in black (n = 9828); observations of *P*. *balsamifera* (n = 755) in grey.

Across the range of sites sampled (Fig. 1), δ^15^N in *P. balsamifera* ranged from −20.6‰ to 8.8‰. Climates for sampled *P. balsamifera* trees spanned ~12 °C of mean annual temperature (MAT) (−1.9 °C to 10.2 °C) and ~2100 mm of mean annual precipitation (MAP) (390–2566 mm). In a model of foliar δ^15^N with MAT, log(MAP), and log[N] as model effects, the addition of *P. balsamifera* observations (n = 755; enlarging the global data set by 7%) exhibited little influence on model estimates compared with using the original global data set (n = 9828) (Table 1). Foliar δ^15^N in *P. balsamifera* generally overlapped with foliar δ^15^N measurements in the global database, though within the restricted range of MAT and MAP exhibited by the *P. balsamifera* samples (Fig. 2). In general, the addition of *P. balsamifera* samples did not alter global patterns found previously: (1) foliar δ^15^N increased with increasing MAT (particularly for MAT >0 °C), (2) foliar δ^15^N decreased with increasing log-transformed MAP, and (3) foliar δ^15^N increased with increasing log-transformed [N] (R^2^ = 0.46 for both models; Table 1). Foliar δ^15^N of the *P. balsamifera* samples alone were generally more weakly related to the effects modeled than were plants from the global data set (Table 1). However, in contrast to the pattern observed for the global dataset, for *P. balsamifera*, foliar δ^15^N decreased with increasing MAT (*P* = 0.0005). *P. balsamifera* foliar δ^15^N showed no relationship with log-transformed MAP (*P* = 0.6). The explanatory ability of mean climate factors and foliar [N] was low (R^2^ = 0.05), leaving considerable small-grain variability remaining in the *P. balsamifera* δ^15^N observations.

**Table 1:** Models of foliar δ^15^N using each of the three data sets.

	Effect	SS^1^	Estimate ± SE^2^	P
Global data; R^2^ = 0.46	Intercept	3721	5.38 ± 0.27	<0.0001
	logMAP^3^	10561	−3.26 ± 0.10	<0.0001
	MAT^4^	65767	0.24 ± 0.003	<0.0001
	Log[N]^5^	24933	7.67 ± 0.15	<0.0001
Global data with *P. balsamifera;* R^2^ = 0.46	Intercept	3885	5.45 ± 0.27	<0.0001
	logMAP	11103	−3.30 ± 0.10	<0.0001
	MAT	70335	0.25 ± 0.003	<0.0001
	Log[N]	25031	7.61 ± 0.15	<0.0001
*P. balsamifera* data; R^2^ = 0.05	Intercept	16.1	−3.51 ± 2.69	0.1916
	logMAP	2.39	0.49 ± 0.97	0.6148
	MAT	115	−0.21 ± 0.06	0.0005
	Log[N]	185	5.17 ± 1.17	<0.0001

^1^Sum of squares. ^2^Standard error. ^3^Base 10 logarithm of Mean Annual Precipitation (mm). ^4^Mean annual temperature (°C). ^5^Base 10 logarithm of foliar nitrogen concentration.

**Figure 3:**
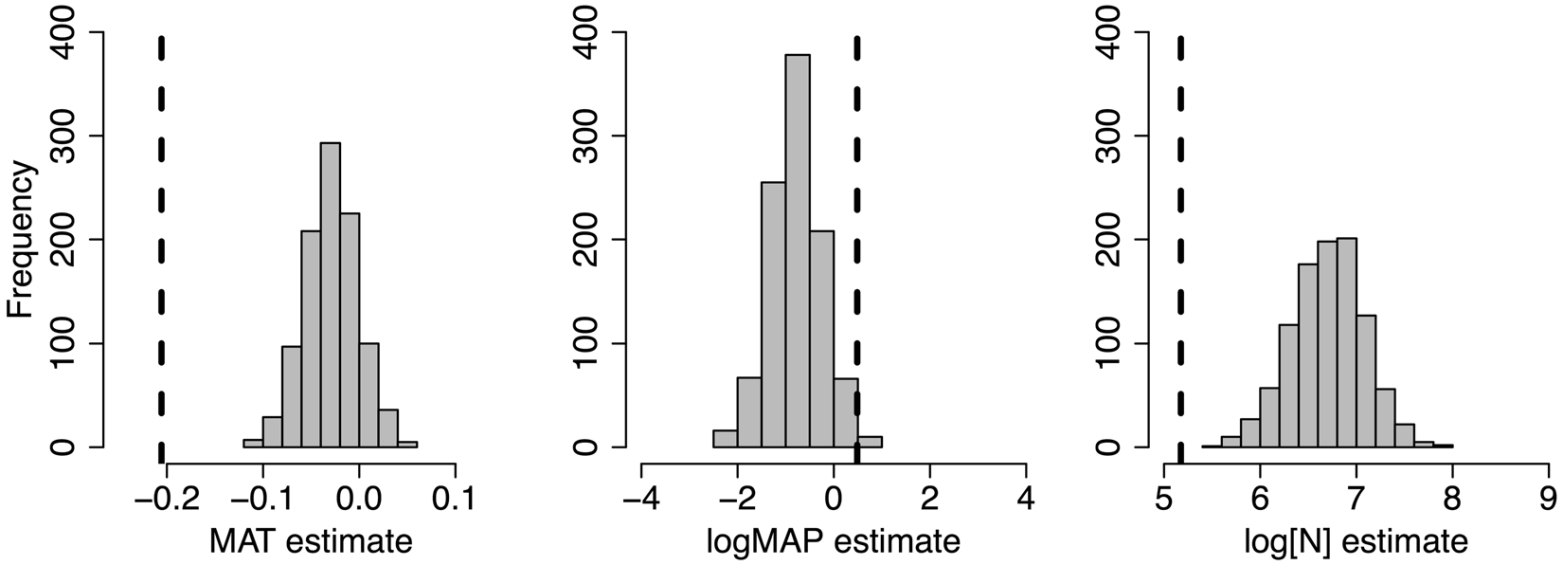
Distribution of model estimates on foliar δ^15^N for MAT, logMAP, and log[N] across 1000 samples of 755 observations in the global N database. Models were constructed as in Fig. 2. Samples were constrained to be within the climate range of *P. balsamifera* samples; the estimates of each effect using only *P. balsamifera* samples is represented by the vertical dotted line (also provided in Table 1).

The unique patterns in relationships between foliar δ^15^N and climate for *P. balsamifera* were also present when subsetting the global data to the same climate envelope as *P. balsamifera*. Repeated random samples of foliar δ^15^N, N, MAT and MAP from the global database within the climate envelope defined by the *P. balsamifera* samples resulted in model estimates for each effect on δ^15^N that were different than the model estimates using *P. balsamifera* alone (Fig. 3). The *P. balsamifera* samples alone resulted in estimates of −0.21‰ °C^−1^ for MAT, 0.49‰ for logMAP, and 5.17‰ for log[N] (Table 1). After accounting for variation imposed by the differing mycorrhizal fungi associations of species, mean estimates resulting from 1000 repeated samples from the global dataset were −0.03 ± 0.001‰ °C^−1^ for MAT, −0.77 ± 0.02‰ for logMAP, and 6.71 ± 0.01‰ for log[N]. These effects of MAT, logMAP, and log[N] were significant (*P* < 0.05) 7.8%, 20.3%, and 100% of the time, respectively. Therefore, relative to the global data set, the effect of MAT on the *P. balsamifera* samples was more negative (*P* < 0.001), the effect of logMAP was removed (i.e., closer to zero; P = 0.6), and the effect of log[N] was reduced, although still significant (P < 0.001).

### Foliar Δ^13^C

**Figure 4:**
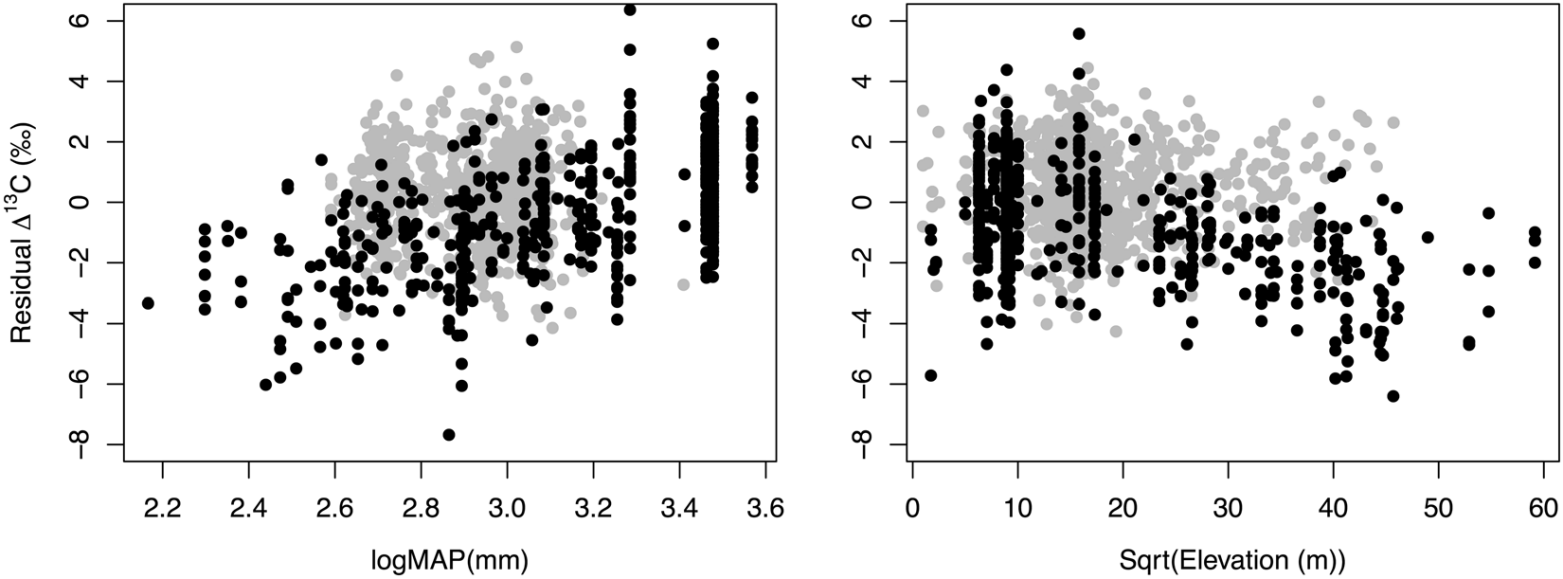
Relationship between residual Δ^13^C and mean annual precipitation (log transformed MAP) and the square root of elevation. For the plot with mean annual precipitation, residual Δ^13^C is calculated relative to a model that only includes the effect of elevation. Similarly, for the plot with elevation, residual Δ^13^C is calculated relative to a model that only includes the effect of precipitation. Global observations from previous work^28^ in black, *P. balsamifera* observations (this study) in grey.

Across the range of sites sampled, foliar Δ^13^C in *P. balsamifera* ranged from 18.2‰ to 25.7‰. Elevation for sampled *P. balsamifera* trees ranged from sea level to 2092 m, which was 60% of the elevation range spanned by the global data set (sea level to 3500 m). After accounting for elevational differences between samples, residual Δ^13^C was plotted against logMAP (Fig. 4) and it was observed that foliar Δ^13^C of *P. balsamifera* tended to be higher than global samples with the same MAP. Mean *P. balsamifera* foliar Δ^13^C was 22.2‰ and mean global foliar Δ^13^C for the same MAP range was 20.6‰ (21.2‰ for angiosperms and 19.1‰ for gymnosperms). Further, after accounting for variation with MAP, high elevation *P. balsamifera* sites exhibited elevated residual Δ^13^C relative to the global samples at the same elevation. As a result, models using *P. balsamifera* samples attribute a smaller fraction of model variance to logMAP and sqrt(elevation) (Sum of Squares values in Table 2) for their effects on Δ^13^C compared with these effects calculated using the global data set.

**Table 2:** Models of foliar Δ^13^C using each of the three data sets.

	Effect	SS^1^	Estimate ± SE^2^	P
Global data	Intercept	258	8.98 ± 0.89	<0.0001
	logMAP^3^	685	4.28 ± 0.26	<0.0001
	sqrt(Elevation)^4^	174	−0.05 ± 0.01	<0.0001
Global data with *P. balsamifera*	Intercept	1435	15.50 ± 0.67	<0.0001
	logMAP	379	2.44 ± 0.20	<0.0001
	sqrt(Elevation)	348	−0.06 ± 0.01	<0.0001
*P. balsamifera* data	Intercept	445	18.40 ± 1.20	<0.0001
	logMAP	23.1	1.36 ± 0.39	<0.0001
	sqrt(Elevation)	13.1	−0.02 ± 0.01	<0.0001

^1^Sum of squares. ^2^Standard error. ^3^Base 10 logarithm of Mean Annual Precipitation (mm). ^4^Square root of elevation (m).

**Figure 5:**
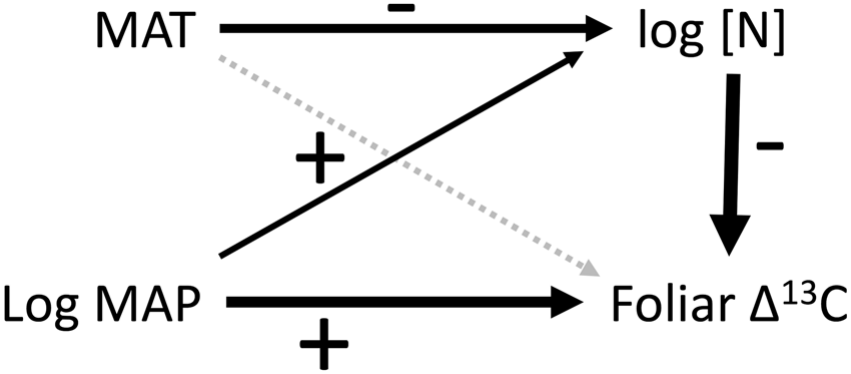
Path diagram illustrating standardized effects (either positive or negative) of mean annual temperature (MAT), mean annual precipitation (log transformed MAP), and foliar N concentration (log transformed) on carbon isotope discrimination in leaves (Foliar Δ^13^C). Arrow widths are proportional to effect sizes; black arrows denote significant effects.

To examine how climate and N availability jointly influence Δ^13^C in *P. balsamifera*, we built a structural equation model (Fig. 5). Results exhibited a positive direct effect of log(MAP) (standardized estimate = 1.74; *P* < 0.001) and a negative direct effect of log(foliar [N]) on Δ^13^C (standardized estimate = −3.23; *P* < 0.001). A positive direct effect of log(MAP) (standardized estimate = 0.07; *P* = 0.025) and a negative direct effect of MAT (standardized estimate = −0.01; *P* = 0.001) on log(foliar [N]) represent indirect effects of climate on Δ^13^C through foliar [N]. The direct effect of MAT on Δ^13^C was not significant, therefore, the only effect of MAT on foliar Δ^13^C is indirectly through foliar [N].

## Discussion

### Foliar δ^15^N response to climate

Through a comparison of the effect of mean climate variables on δ^15^N between a global data set, including hundreds of different plant species, and a single widely distributed species, *P. balsamifera*, we found that broad scale patterns in the isotopic composition of foliar tissues observed globally is largely weakened when observed in a single species. Across global gradients in climate, foliar δ^15^N measured in a wide variety of species has been shown to correlate with MAT, MAP, and [N], and mycorrhizal fungi association^10^. Demonstrating the same for a single species is often difficult due to the limited climatic variability encompassed by the range of most species. For the widely distributed *P. balsamifera*, we found weak, but significant effects of MAT and foliar [N], but not MAP (Table 1). The small effect of climate on foliar δ^15^N in *P. balsamifera* is consistent with the idea that the global patterns in foliar δ^15^N published previously were a result of geographic variation in species presence collinear with geographic variation in climate. However, the more limited climate range occupied by *P. balsamifera* complicates this interpretation.

Random samples from the global database within the climate envelope defined by the range of *P. balsamifera* (a sample pool of 1937 observations) exhibited significant effects of MAT, MAP and [N] on δ^15^N 7.8%, 20.3%, and 100% of the time (respectively, across 1000 resampling). Comparing global and *P. balsamifera* samples, the largest difference is the effect of MAT, which had a significant negative effect on foliar δ^15^N in *P. balsamifera*. These patterns afford some speculation as to the mechanisms behind global foliar δ^15^N patterns. For example, increasing foliar δ^15^N with increasing MAT between −0.5 and 30 °C is thought to represent a gradient in the dominant pathway responsible for the loss of N from ecosystems, from DON leaching (a low fractionation pathway) at low MAT to denitrification (a high fractionation pathway) at high MAT^10^. For *P. balsamifera*, occupying a MAT range from −1.9 to 10.2 °C, the enrichment of ^15^N decreased with increasing MAT, which is counter to the broad global pattern, but consistent with the 7.8% of models from the global database that were significant across this temperature range. For *P. balsamifera*, therefore, one possible interpretation is that N availability decreases with increasing temperature. However, it is also possible that *P. balsamifera* becomes more reliant on NO_3_^-^ as opposed to NH_4_^+^ with increasing temperature or is more likely to occupy sites with lower nutrient availability, leading to a small decline in foliar δ^15^N with increasing temperature^22^.

After accounting for MAT, the effect of MAP on foliar δ^15^N was weaker for *P. balsamifera* than for the global samples. The global pattern of decreasing foliar δ^15^N with increasing MAP has been explained as a response to higher gaseous N loss in more xeric environments^36^ and low N availability (potentially due to high nitrate leaching) in wet environments^23, 37^. For the relatively mesic environments studied here, there was no effect of MAP, suggesting that precipitation amount is potentially not a driver of denitrification for the sites inhabited by *P. balsamifera* across this gradient. This might be a consequence of the habitat preferences of *P. balsamifera*, which is for wet locations such as riparian zones and the edges of wetlands, which might act to normalize the effect of precipitation on δ^15^N across its range.

The effect of foliar [N] on foliar δ^15^N was smaller for *P. balsamifera* than it was for the global samples. After accounting for MAT and MAP, for every 1% increase in foliar [N] δ^15^N increased 5‰. Considering a foliar δ^15^N range of ~30‰, N availability appears to be highly variable in *P. balsamifera*, possibly associated with variation in productivity across its range. However, it is also clear that there is considerable site-to-site variability in N availability unexplained by the mean climate factors studied here. This variability is likely due to any number of factors, such as site soil moisture conditions, variable anthropogenic inputs, and site disturbance history^19^.

### Joint influence of precipitation and foliar nitrogen on Δ^13^C

Adding *P. balsamifera* samples to the global database reduced the effect of MAP and elevation on foliar Δ^13^C. The effect of elevation on Δ^13^C has been discussed as related to temperature, the partial pressure of atmospheric CO_2_ or O_2_, irradiance, or edaphic factors^28^, but is still a poorly understood process^33^. There is also a trend towards greater dominance of evergreen gymnosperms at higher elevations, which have lower Δ^13^C values than angiosperms. Diefendorf *et al*.^28^ calculated that at the site level, evergreen gymnosperms on average exhibited 2.7‰ lower Δ^13^C than deciduous angiosperms. Therefore, not accounting for plant functional type in models will likely exaggerate the effect of elevation on Δ^13^C, but this is just one possible influence of elevation on Δ^13^C. By considering only *P. balsamifera* samples we explicitly account for interspecific variation, effectively removing most of the effect of elevation on Δ^13^C (Table 2). Beyond all coming from the same species, the samples of *P. balsamifera* also have the potential to normalize for habitat conditions typical of locations occupied by this species. Therefore, the reduced sensitivity of *P. balsamifera* Δ^13^C to elevation lends support to the idea that species turnover and variation in the site conditions encountered along elevation gradients explain the mechanisms behind the effect of elevation on Δ^13^C more than atmospheric differences.

The positive effect of MAP on Δ^13^C is understood as a result of reduced stomatal conductance at low MAP, which decreases water loss through transpiration more effectively than it decreases assimilation, thus increasing water use efficiency. The fact that we see this effect in *P. balsamifera* samples is consistent with the idea that trees from dryer locations exhibit greater water use efficiency. However, δ^15^N, and therefore N availability, trended lower with increasing MAP (Fig. 2), which has a similar effect on Δ^13^C. This is commonly seen in terrestrial plants: plants with high N availability exhibit increased assimilation, which reduces carbon discrimination (Δ^13^C) at the same stomatal conductance^34^. For a single tree species with presumably similar nitrogen and water acquisition strategies across its range, the effects of increased N availability and reduced stomatal conductance at low precipitation sites have the effect of reducing Δ^13^C. We see these direct and indirect effects on Δ^13^C in our structural equation model (Fig. 5), which shows significant direct effects of foliar [N] and logMAP on Δ^13^C. MAT only influences Δ^13^C indirectly through its effect on foliar [N]. The explanatory power of this model using *P. balsamifera* samples alone is small, however, suggesting that differences among species dominate the global pattern.

In summary, we generally found different responses to climate in plant foliar chemical traits measured in a single widely distributed species than the same measured in many species distributed globally. Foliar chemical traits in *Populus* spp. have been found to vary across distinct genotypes^38–40^. However, we find that this variation is not always congruent with variation due to species and plant functional type distributions. This is not to say that global variation in foliar isotopes, driven by species turnover across climate and elevational gradients, is in some way misleading. For many applications (e.g., interpreting paleo records^26^), global variation across many species is exactly what is needed. By studying a single species, we control for one source of variability on foliar isotopes, providing insight into mechanisms that are unique to that species or the habitat it occupies. Specific to δ^15^N, measurements of *P. balsamifera* exhibit a negative effect of MAT that was not measured across a similar climate range in globally distributed foliar samples. Among the possible interpretations is that N availability to these trees is reduced along the southern range edge of this species. Likewise, for foliar Δ^13^C, the effect of elevation was reduced in *P. balsamifera*, which suggests that the habitat’s *P. balsamifera* occupies somehow normalize for the effect of elevation on foliar Δ^13^C. It is difficult to use these observations of *P. balsamifera* to generalize to other species or mechanisms driving geographic variability. However, it seems clear that global variation in foliar δ^15^N and Δ^13^C is driven by variation between species, each with its own unique relationships to climate and elevation. The importance of this finding will only be realized as further research attempts rectify intraspecific and interspecific isotopic patterns in a variety of species, ideally leading to stronger generalities of how climate affects resource availability.

## Methods

*Populus balsamifera* is a dominant deciduous tree across most of the boreal forest of North America (Fig. 1). In the southern edge of its extent (throughout the forests of New England and the upper Midwest, USA), it occurs in low density, preferring wetter sites and sites exhibiting recent disturbance. *P. balsamifera* generally exhibits ectomycorrhizal associations, however, there is some variation in mycorrhizal association across the *Populus* genus^41^. In the northern portion of its range, it is often the only deciduous tree or is codominant with *Populus tremuloides*. Studies of genetic variation in *P. balsamifera* show that the structure of population diversity reflects broad patterns of geographic expansion following the last glacial maximum, with three main demes evident located in the northwest, center, and eastern portions of its range^42, 43^. If intraspecific differences control variation in chemical foliar traits in *P. balsamifera* then we expect δ^15^N and Δ^13^C values to cluster into similar geographic regions. However, such patterns could also be superseded by global patterns in foliar traits driven by environmental gradients^10^.

We sampled 755 specimens of *P. balsamifera* during the 2015 growing season. Samples were spaced >20 km apart along navigable roads to facilitate access, which also lead to a bias towards preferentially sampling within ~100 m of roads and across the southern range edge of this species (where there is greater access) (Fig. 1). While it is known that traffic emissions can influence foliar δ^15^N in road-side plants^17, 44^, we had no data on traffic patterns and ultimately chose not to investigate this effect in our samples. If an effect of traffic is present in our data, we suspect it would not be collinear with climate or elevation. For each sample, a hand-held global positioning system receiver was used to record the geographical location (including elevation) of the sample with a locational uncertainty of <30 m. Sites spanned nearly 23 degrees of latitude (39.87°N to 62.74°N) and 90 degrees of longitude (60°W to 150°W) (Fig. 1). Foliar samples were selected from sun leaves on each tree, acquired via a pruning pole or equivalent. Each sample was placed in a plastic bag with 10g of silica gel to act as a desiccant during transport to the laboratory where foliar samples were then dried at 60 **°**C for 48 hours. Leaves were homogenized, and approximately 2 mg of foliar tissue from each sample was analyzed for [C], [N], δ^13^C and δ^15^N using a Carlo Erba NC2500 elemental analyzer (CE Instruments, Milano, Italy) interfaced with a ThermoFinnigan Delta V + isotope ratio mass spectrometer (IRMS; Bremen, Germany). A MgClO_4_ trap was used to remove water vapor prior to the transfer of sample gases to the IRMS. The δ^13^C and δ^15^N data were normalized to the VPDB and AIR scales, respectively, using a two-point normalization curve with internal standards calibrated against USGS40 and USGS41. The long-term analytical precision (1σ) of an internal leaf standard analyzed alongside samples was 0.28‰ for δ^13^C and 0.24‰ for δ^15^N. Carbon isotope discrimination against ^13^C (Δ^13^C) was calculated according to Farquhar *et al*.^29^ as:

1 $${{\rm{\Delta }}}^{13}C=\frac{{\delta }^{13}{C}_{air}-{\delta }^{13}{C}_{plant}}{1+{\delta }^{13}{C}_{plant}/1000}$$

where the δ^13^C_air_ value used was the δ^13^C measured in the atmosphere at Mauna Loa, Hawaii in June 2015 (δ^13^C_air_ = −8.6‰). Elevated Δ^13^C signifies greater increases in intercellular CO_2_ concentrations (C_i_) than atmospheric CO_2_ concentrations (C_a_), which is caused by increased isotopic fractionation due to either greater stomatal conductance and/or lower photosynthetic assimilation rates in C_3_ plants^45^. Foliar [N] was log transformed to achieve a normal data distribution for comparison with δ^15^N and climate parameters.

We attained gridded climate data from DAYMET 1980–2015, which is an interpolated product providing minimum and maximum temperature and precipitation totals at daily intervals^46, 47^. Daily mean temperatures were calculated as T_mean_ = (T_min_ + T_max_)/2. From the daily means, mean annual temperature (MAT) was calculated by first calculating annual means 1980–2015, which were then averaged. Similarly, mean annual precipitation (MAP) was calculated as the mean annual sum of daily precipitation, 1980–2015. MAP was log_10_ transformed to achieve a normal data distribution. The square root of elevation was calculated to achieve a normal data distribution.

A global data set of foliar [N] and δ^15^N (n = 9828) was acquired from Craine *et al*.^10^. Therefore, the number of measurements of δ^15^N of *P. balsamifera* was 7.7% of the global data set. The global data set included data on mean annual temperature (MAT), mean annual precipitation (MAP) both extracted from^48^, geographic location, and species. The global data set represented foliar samples from across the full spectrum of global climates, whereas the *P. balsamifera* data were constrained to climate conditions in northern North America. A global data set of foliar Δ^13^C in trees (n = 570) was acquired from Diefendorf *et al*.^28^. These data represent species mean Δ^13^C at each site (between 1 and 227 individuals were sampled, n = 3310), rather than individual observations, thus removing within-species variability at each site. This data set included site MAP and elevation.

After completing a general survey of the data and evaluating the geographic and climatic range represented by the data, we followed procedures described by Craine *et al*.^10^ and Diefendorf *et al*.^28^ to build models relating δ^15^N and Δ^13^C to climate factors. We combined the global and *P. balsamifera* datasets and built ordinary least squares models of foliar δ^15^N and Δ^13^C with a selection of MAT, logMAP, log[N] (for δ^15^N), and logMAP and sqrt(elevation) (for Δ^13^C) as model effects. Models using the (1) global data sets, (2) the global data sets combined with the new *P. balsamifera* observations, and (3) the *P. balsamifera* observations alone were compared to investigate the impact of the addition of the *P. balsamifera* samples on the global relationships. To facilitate visualization, for each of the models using the combined data sets, we iteratively built models that left one predictor out and then plotted the model residuals against the remaining predictor. For δ^15^N, the global data set contained a sufficient number of observations within the climate range exhibited by the *P. balsamifera* data (n = 1937) to estimate the effects of MAT and MAP on foliar δ^15^N across samples spanning a similar range in these predictors. Therefore, we repeatedly (x1000) sampled 755 random observations from the global database constrained to have the same minimum and maximum climate values as exhibited by the *P. balsamifera* samples. To account for variation in mycorrhizal association between the many species represented in the global foliar δ^15^N database, we used normalization factors published by Craine *et al*.^10^ and applied an offset to each measurement so that mean foliar δ^15^N was the same for ericoid (+2.7‰), ectomycorrhizal (0‰), arbuscular (−1.2‰), and non-mycorrhizal (−3.2‰) plants. Using the 755 selected observations, we ran a model with MAT, logMAP, and log[N] as model effects and recorded the estimates for each predictor. We then repeated the model on each of the 1000 random resampled data sets and calculated the mean and standard deviation of the resulting model estimates for comparison with estimates of the same predictors on *P. balsamifera* foliar δ^15^N alone. There were insufficient samples in the global database to complete the same random resampling procedure for Δ^13^C.

The impact of global patterns of N availability and resulting foliar N on water relations in plants (and foliar Δ^13^C) is an outstanding question, particularly among widely distributed plant species that exist across a wide range of climate conditions. However, such relationships have rarely been evaluated for a single, widely distributed species. Therefore, we explored the importance of direct and indirect effects of MAT, MAP, and log[N] on Δ^13^C using a structural equation model (SEM). The model we used contained no latent variables, but did include all possible pathways between climate variables and the foliar chemical variables. This model was implemented in the R programing language using the Lavaan package version 0.5–22^49^.
